# Highly-stable write-once-read-many-times switching behaviors of 1D–1R memristive devices based on graphene quantum dot nanocomposites

**DOI:** 10.1038/s41598-018-30538-y

**Published:** 2018-08-13

**Authors:** Sihyun Sung, Chaoxing Wu, Hyun Soo Jung, Tae Whan Kim

**Affiliations:** Department of Electronics and Computer Engineering, Hanyang University, Seoul, 04763 South Korea

## Abstract

One diode and one resistor (1D–1R) memristive devices based on inorganic Schottky diodes and poly(methylsilsesquioxane) (PMSSQ):graphene quantum dot (GQD) hybrid nanocomposites were fabricated to achieve stable memory characteristics. Current-voltage (I-V) curves for the Al/PMSSQ:GQDs/Al/p-Si/Al devices at room temperature exhibited write-once, read-many-times memory (WORM) characteristics with an ON/OFF ratio of as large as 10^4^ resulting from the formation of a 1D–1R structure. I-V characteristics of the WORM 1D–1R device demonstrated that the memory and the diode behaviors of the 1D–1R device functioned simultaneously. The retention time of the WORM 1D–1R devices could be maintained at a value larger than 10^4^ s under ambient conditions. The operating mechanisms of the devices were analyzed on the basis of the I–V results and with the aid of the energy band diagram.

## Introduction

The enhancement of the electrical characteristics for memristive devices fabricated utilizing hybrid nanocomposites has been intensively investigated owing to their having excellent advantages of low cost, high flexibility, simple fabrication, and low power consumption^1–3^. When memristive devices are fabricated utilizing inorganic/organic nanocomposites, charge-storage and matrix materials are typically combined by using a spin-coating deposition^4^. Moreover, metallic Au, Al, Ag, and Cu nanoparticles are extensively used as charge-trapping materials^4–6^. However, because almost all metallic nanoparticles are not only expensive but also unstable at high temperatures, nanocomposites containing metallic nanoparticles have inherent problems for practical applications in memory devices. Graphene quantum dots (GQDs), which are included in the category of the ultrafine graphene family, have emerged as excellent charge-trapping materials for potential applications in memristive memory devices because of their unique chemical inertness, low toxicity, and large work function^7^. Furthermore, GQDs with a nanoscale size contain significant edge effects and strong quantum confinement, especially in nanoscale devices^8^. The crystallographic orientation of the graphene edges significantly influences the electronic properties of the GQDs, including a Coulomb blockade and a mobility gap^9^. In addition, poly(methylsilsesquioxane) (PMSSQ) materials have attracted much attention due to their superior physical properties of low-dielectric constant, low moisture absorption, excellent thermal stability, excellent mechanical hardness, and simple synthesis^10–12^. Therefore, hybrid nanocomposites based on GQDs with a remarkable charge-storage capability embedded in a polymer layer with a low-dielectric constant are very effective in serving as the active layer in memristive devices^13^.

Cross-talk interference between memristive cells can originate from leakage current paths through neighboring cells in cross-bar array or an excess of current, may cause electrical misreading of the device^14–17^. This phenomenon in memristive devices disturbs the reading process of the selected cell and must be eliminated before such devices can be used for practical applications. An effective method for eliminating the cross-talk is to connect a rectifying diode or selector to each cell^18,19^. Recently, a write-once, read-many-times (WORM) memory with a diode, in which undesired cross-talk was prevented, was demonstrated^20^. The architecture of one diode and one resistor (1D–1R) can improve reading accessibility in an integrated memory array structure^21–23^. The 1D–1R architecture is preferred in terms of integration because it occupies less area. Furthermore, the design and the fabrication of 1D–1R devices are relatively very simple.

## Methods

The WORM 1D–1R devices contain an active layer of GQDs embedded in a PMSSQ layer on an p-Si/Al Schottky diode. Firstly, The PMSSQ was prepared first by mixing de-ionized (DI) water, n-butanol (Aldrich), and trimethoxymethylsilane [CH_3_Si(OCH_3_)_3_] (Aldrich) in a weight ratio of 1:10:4. The mixture underwent ultrasonic processing for 24 h at 60 °C. Then, the GQD solution (ACS MATERIAL) was added to the PMSSQ solution in a weight ratio of 0, 10, or 20%, followed by an ultrasonic process for 2 h at room temperature^13^. The p-type (100) Si substrates (containing native oxide layers) with a resistivity of 1–10 Ω were cleaned ultrasonically in acetone, methanol, and de-ionized (DI) water for 30 min each. After the chemically-cleaned p-Si substrates had been dried by using N_2_ gas with a purity of 99.99%, middle Al electrodes, each with a thickness of 70 nm, were thermally evaporated at a pressure of 1 × 10^−6^ Torr. Then, the memristive devices with GQDs embedded in an insulating PMSSQ layer were fabricated on Al/p-Si substrate. The PMSSQ:GQDs thin layers were formed on Al/p-Si substrates by using a spin-coating method with spin-coating speeds of 500 rpm for 3 s, 1000 rpm for 5 s, 3000 rpm for 30 s, 1000 rpm for 5 s, and 500 rpm for 3 s in series at room temperature. Then, the devices were annealed at 140 ° for 1 h. The top Al electrodes, each with a thickness of 180 nm and a diameter of 1 mm, were deposited on the PMSSQ:GQDs layer by using thermal evaporation through a metal mask at a system pressure of 1 × 10^−6^ Torr. Finally, to fabricate the p-Si/Al Schottky diodes, we deposited bottom Al electrodes, each with a thickness of 200 nm, on the back side of the Si substrate by using thermal evaporation at a system pressure of 1 × 10^−6^ Torr.

The structural properties of the Al/:PMSSQ:GQDs/Al/p-Si/Al devices were characterized by using scanning electron microscopy (SEM, NOVA NANO SEM 450). All electrical measurements on the devices were performed by using a semiconductor characterization system (Keithley 2400) at 300 K. The bottom Al electrode was grounded during the memory-effect measurements. When the performances of the resistive memory were measured, the voltage was applied to the top Al electrode and the middle Al electrode, when those of the diode were measured, the voltage was applied to the middle Al electrode and the bottom Al electrode, and when those of the 1D–1R device were measured, the voltage was applied to the top Al electrode and the bottom Al electrode.

## Results and Discussion

**Figure 1:**
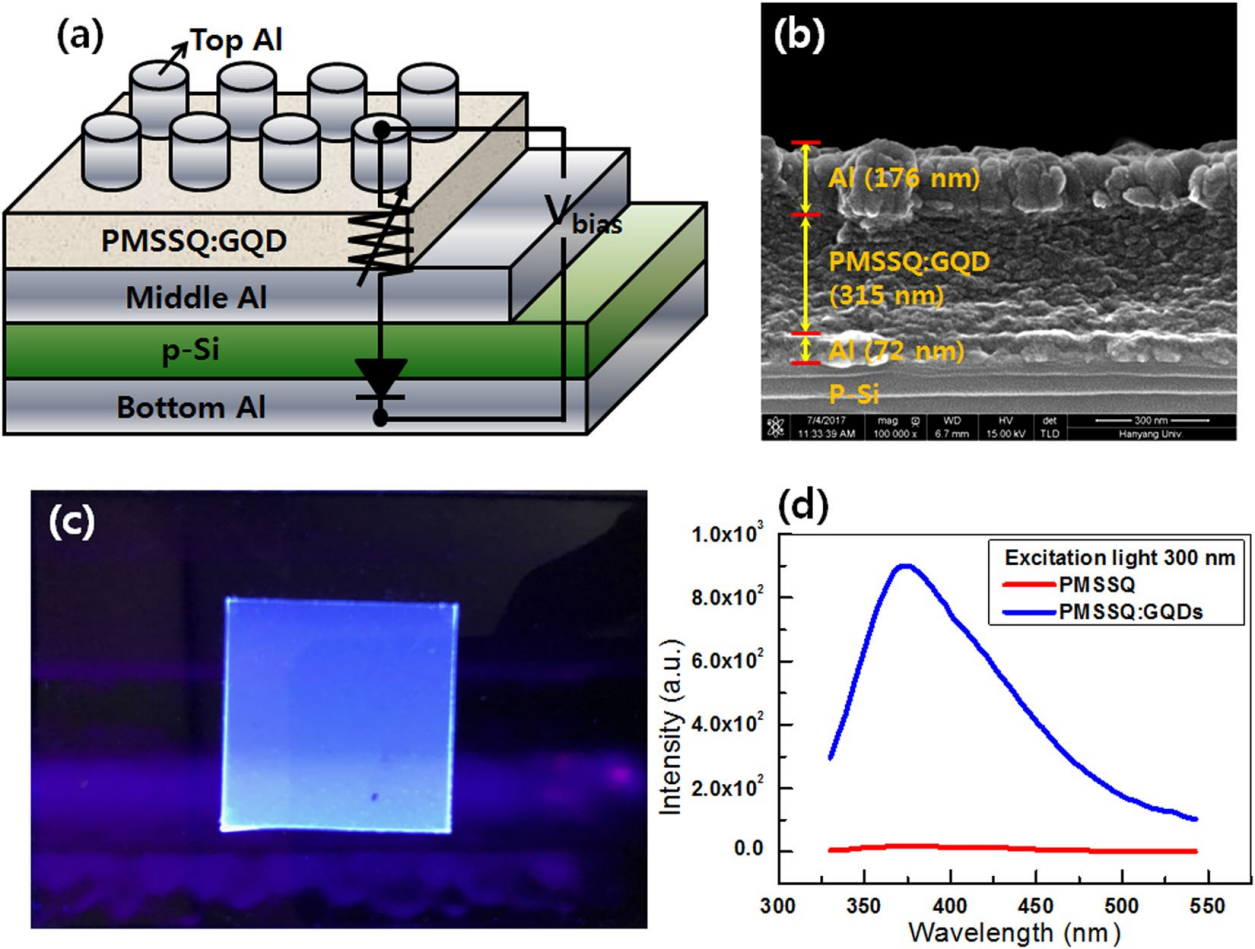
**(a)** Schematic diagram of the Al/PMSSQ:GQDs/Al/p-Si/Al devices. **(b)** Scanning electron microscopy image of the Al/PMSSQ:GQDs/Al/p-Si/Al device. **(c)** Optical image of the PMSSQ:GQDs thin-film under 254-nm UV illumination exhibited bright blue emission. **(d)** Photoluminescence spectra from the PMSSQ and the PMSSQ:GQDs thin-film under 300-nm exciting light.

Figure 1 shows a (a) schematic diagram and a (b) cross-sectional SEM image of the 1D–1R device with an Al/PMSSQ:GQDs/Al/p-Si/Al structure. The structure of the device consisted of a PMSSQ active layer containing GQDs and a Al/p-Si layer, with those two layers separating the top and the bottom Al electrodes. The thicknesses of the top Al electrode, the PMSSQ:GQDs nanocomposite film, and the Al layer on the p-Si were approximately 176, 315, and 72 nm, respectively. Figure 1(c) demonstrates that the PMSSQ:GQDs thin film exhibits a bright blue emission under 254-nm UV illumination. Figure 1(d) presents the photoluminescence spectra from the PMSSQ and the PMSSQ:GQDs thin films under excitation by 300-nm light; the results are indicative of the existence of GQDs in the deposited film.

**Figure 2:**
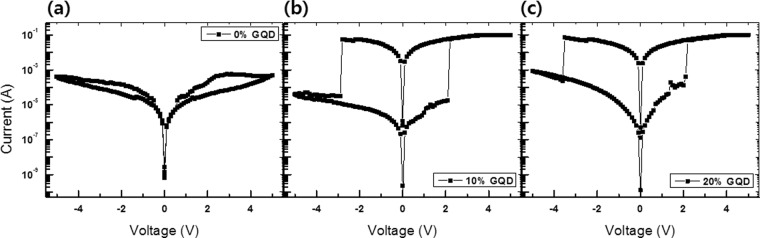
Current-voltage curves for the Al/PMSSQ:GQDs/Al/p-Si/Al devices with GQD concentrations of **(a)** 0, **(b)** 10, and **(c)** 20%.

Figure 2 shows I-V curves for the PMSSQ resistive memory devices with GQD (Al/PMSSQ:GQDs/Al) concentrations of (a) 0, (b) 10, and (c) 20%. The bottom Al electrode was grounded, and the voltage was swept from −5 to  t5 V; thus, both charge trapping and de-trapping currents occurred. Figure 2(a) shows the I-V curves for the devices with only a PMSSQ active layer. Even though the devices with GQD concentrations of 0% showed slight memory characteristics, their electrical characteristics were unstable. The I-V curves for the Al/PMSSQ:GQDs/Al devices with a GQD concentration of 10% demonstrated bipolar characteristics, as shown in Fig. 2(b). When the voltages applied to the device were varied from 0 → 2.1 (OFF) → 5 (ON) → −2.8 (ON) → −5 (OFF), the corresponding currents of the ON and the OFF states at 1 V were 2.29 × 10^−2^ and 3.48 × 10^−6^ A, respectively. Figure 2(c) presents the I-V curves for the devices containing an active layer with GQDs embedded in a PMSSQ layer at a GQD concentration or 20%. The hysteresis behaviors observed in the I-V curves for those devices, on a log scale, were similar to those of the devices fabricated with a GQD concentration of 10%. The ON and the OFF currents at 1 V were 2.4 × 10^−2^ and 2.36 × 10^−5^ A, respectively. The ON/OFF ratio of the devices with a GQD concentration of 20% was smaller than that of the devices with a GQD concentration of 10%.

Even though the structure of the device is symmetrical, its electrical behaviors are asymmetric. Because of the fabrication process and post-treatment, the top-Al/PMSSQ interface is actually different from the bottom-Al/PMSSQ interface. For the bottom-Al/PMSSQ interface, the PMSSQ solution is spin-coated onto the Al electrode, followed by annealing at 140 °f for 1 h. However, for the top-Al/PMSSQ interface, the Al electrodes are directly deposited on the surface of the PMSSQ. Thus, the two interfaces are different. According to the I-V curves in Fig. 2 and the working mechanism based on charging/discharging of traps, which will be discussed later, electrons seem to be more easily injected into the active layer from the middle Al electrode.

**Figure 3:**
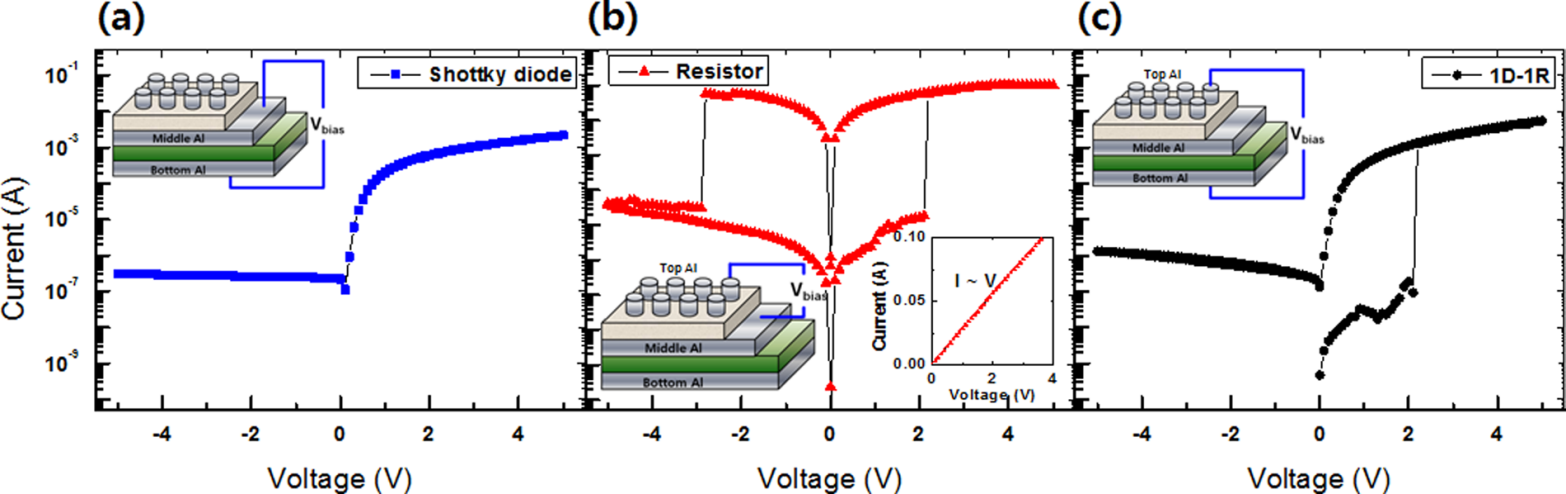
Current-voltage curves and schematic diagram of electrical contact of the Al/PMSSQ:GQDs/Al/p-Si/Al devices (left insets) of the **(a)** p-Si/Al Schottky diode, **(b)** Al/PMSSQ:GQDs/Al device, and **(c)** Al/PMSSQ:GQDs/Al/p-Si/Al 1D–1R device.

Figure 3 shows the I-V curves for the (a) Schottky diode, the (b) resistive memory, and the (c) 1D–1R devices. The I-V curve of the p-Si/Al junction exhibited nonlinear diode characteristics, as shown in Fig. 3(a). Before the performances of the p-Si/Al diodes were measured, the edge of the PMSSQ:GQD thin layer that had been spin-coated was removed using acetone to allow contact with the middle Al electrode in the 1D–1R devices. The currents for positive applied voltages between −5 and +5 V were much higher than those under negative applied voltages. The Al/p-Si/Al Schottky diode presented excellent rectifying properties with a high rectification ratio. A Schottky junction can be formed by directly evaporating Al on a p-Si substrate; furthermore, an Ohmic junction can be formed by evaporating Al on a p-Si substrate, followed by thermal annealing at 550 °u for 20 min^22^. In our work, based on the diode’s rectifying I-V characteristics, we believe that an Ohmic junction was formed on the front side of the p-Si substrate while a Schottky junction was formed on the back side of the p-Si substrate. Worth noting is that when we prepared the PMSSQ layers, the device with a structure of PMSSQ:GQDs/Al/p-Si was annealed at 140 °l for 1 h, which is the reason for an Ohmic junction being formed on the front side of the p-Si substrate.

The I-V curves for the resistive memory devices fabricated utilizing PMSSQ:GQDs nanocomposites exhibited bipolar characteristics, as shown in Fig. 3(b). The electrical characteristics of the devices with a GQD concentration of 10% demonstrated the largest memory margin, as shown Fig. 2(b). The I-V characteristics of a combined 1D–1R device with a structure of Al/PMSSQ:GQDs/Al/p-Si/Al show that the memory and the diode characteristics of the 1D–1R device functioned simultaneously, as shown in Fig. 3(c). The reversible switching characteristics of unipolar memory devices are attributed to the rectifying properties of the diodes. While the current in the 1D–1R devices dramatically increased at a threshold voltage of 2.2 V in the range of positive sweep voltages, indicative of a transition from the OFF to the ON states, no current flow of any significant size was measured under negative applied voltages. However, the current in the 1D–1R devices with a structure of Al/PMSSQ:GQDs/Al/p-Si/Al under an applied positive voltage was smaller than that of the resistive memory devices with a structure of Al/PMSSQ:GQDs/Al. The ON/OFF current ratios of the resistive memory and the 1D–1R devices at 1 V were 88 and 1.16 × 10^2^, respectively. Because the p-Si/Al diode layer with a large resistivity affects the electrical characteristics of the 1D–1R devices, the decrease in the OFF current of the 1D–1R devices originates from the existence of the p-Si/Al Schottky diode, resulting in the prevention of the crosstalk effect due to the role played by the diode.

**Figure 4:**
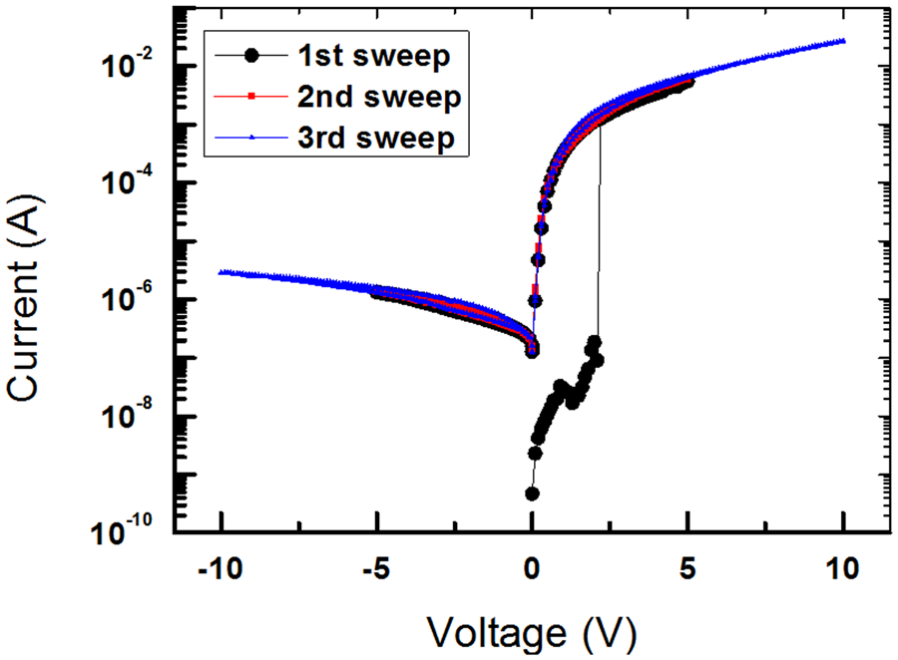
Current-voltage curves of the Al/PMSSQ:GQDs/Al/p-Si/Al device during three voltage sweeps.

Figure 4 shows the I-V curves for the WORM 1D–1R devices during three voltage sweeps. The first and the second sweeps were done in the voltage range between −5 and 5 V. During the first voltage sweep, the devices initially maintained an OFF state at low voltages. When the applied voltage reached 2.2 V, the current rapidly increased from 9.17 × 10^−8^ to 1.26 × 10^−3^ A, indicative of a state transition in the memristive devices from an OFF state to an ON state. After the stage transition had occurred, the current in the devices gradually increased from 1.3 × 10^−3^ to 5.5 × 10^−3^ A with increasing applied bias voltage between 2.2 and 5V. The current ratio between the ON and the OFF states for the 1D–1R devices at a read bias voltage of 1V was as large as approximately 1.09 × 10^4^, which was large enough to decrease significantly the misreading probability from the memory identification of the devices. After the transition from the OFF to the ON states, the device stayed in the ON state, as shown in the curves for the subsequent voltage sweeps from 5 to 0V. The decrease in the current of the devices when the voltage was swept from 0 to −5V could be attributed to the rectifying properties of the p-Si/Al diode. During the second voltage sweep, the devices showed WORM characteristics. The ON state of the devices only appeared in the voltage range between 0 and 5V. The electrical characteristics of the devices were similar in the negative applied voltage range due to the existence of the p-Si/Al diode, which is the same behavior as was observed in the I-V curves for the devices during the first voltage sweep. The third sweep was performed in the voltage range between -10 and 10V to observe the electrical characteristics at large applied voltages. The behaviors of the electrical characteristics during the third sweep stage were very similar to those during the first and the second sweeps. No resistive switch phenomena occur. The reader should note that the introduction of a diode to the memory device leads to the write-once limitation. Thus, the proposed 1D–1R memory devices can only be used in an electronic system that requires a WORM memory.

**Figure 5:**
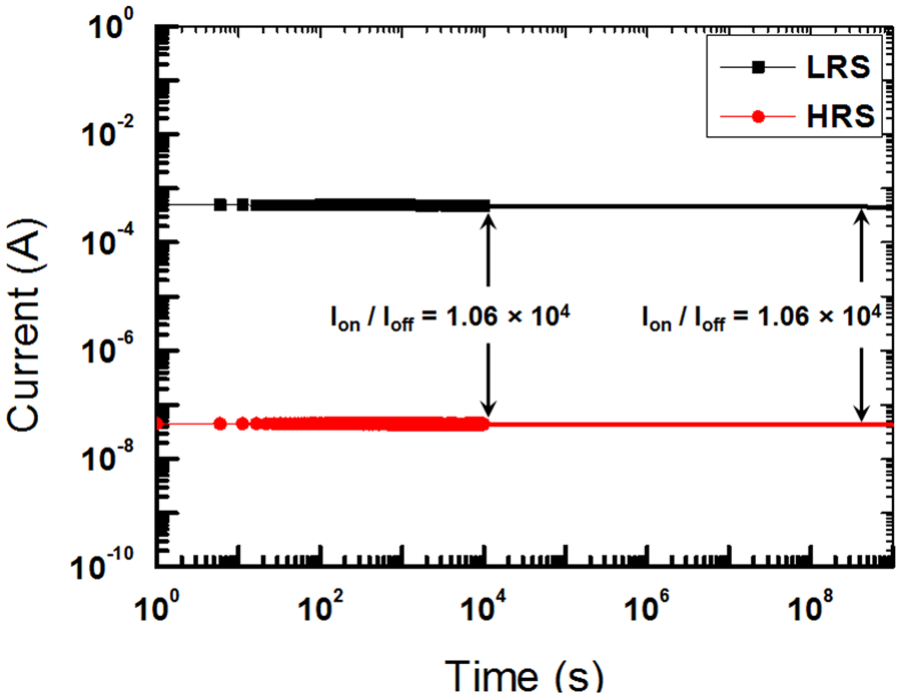
Retention characteristics for the Al/PMSSQ:GQDs/Al/p-Si/Al device at a reading voltage of 1V.

The retention abilities of the WORM 1D–1R devices were determined by measuring the performances of the OFF and the ON states of the devices under ambient conditions. After the fixed SET voltage had been applied to the devices, the currents corresponding to the ON and the OFF states were measured at a reading voltage of 1 V. As shown in Fig. 5, while the current in the ON state of the devices was about 4.73 × 10^−4^ A, that in their OFF state was approximately 4.45 × 10^−8^ A. The current-time (I-t) data show that an ON/OFF current ratio of 10^4^ was maintained for retention times longer than 1 × 10^4^ s No significant degradation in behavior was observed during a retention time of 10^4^ s, indicating that the electrical characteristics of the devices were stable for at least 10^4^ s. As discussed in ref.^23^, the resistance states (ON and OFF states) should not be stable during read-out as the structure is a 2-terminal device^24^. However, the ON and the OFF states were very stable for our device. The possible mechanism for explaining this difference is that the GQDs are acting as electron-trapping sites in our device. Thus, an electron trapped in the insulating layer is more stable under a small applied voltage, such as the reading voltage. When the I-t curves for the devices were extrapolated to 10 years, they converged to an ON/OFF ratio of 1.06 × 10^4^, which is indicative of the long-time stability of the devices.

**Figure 6:**
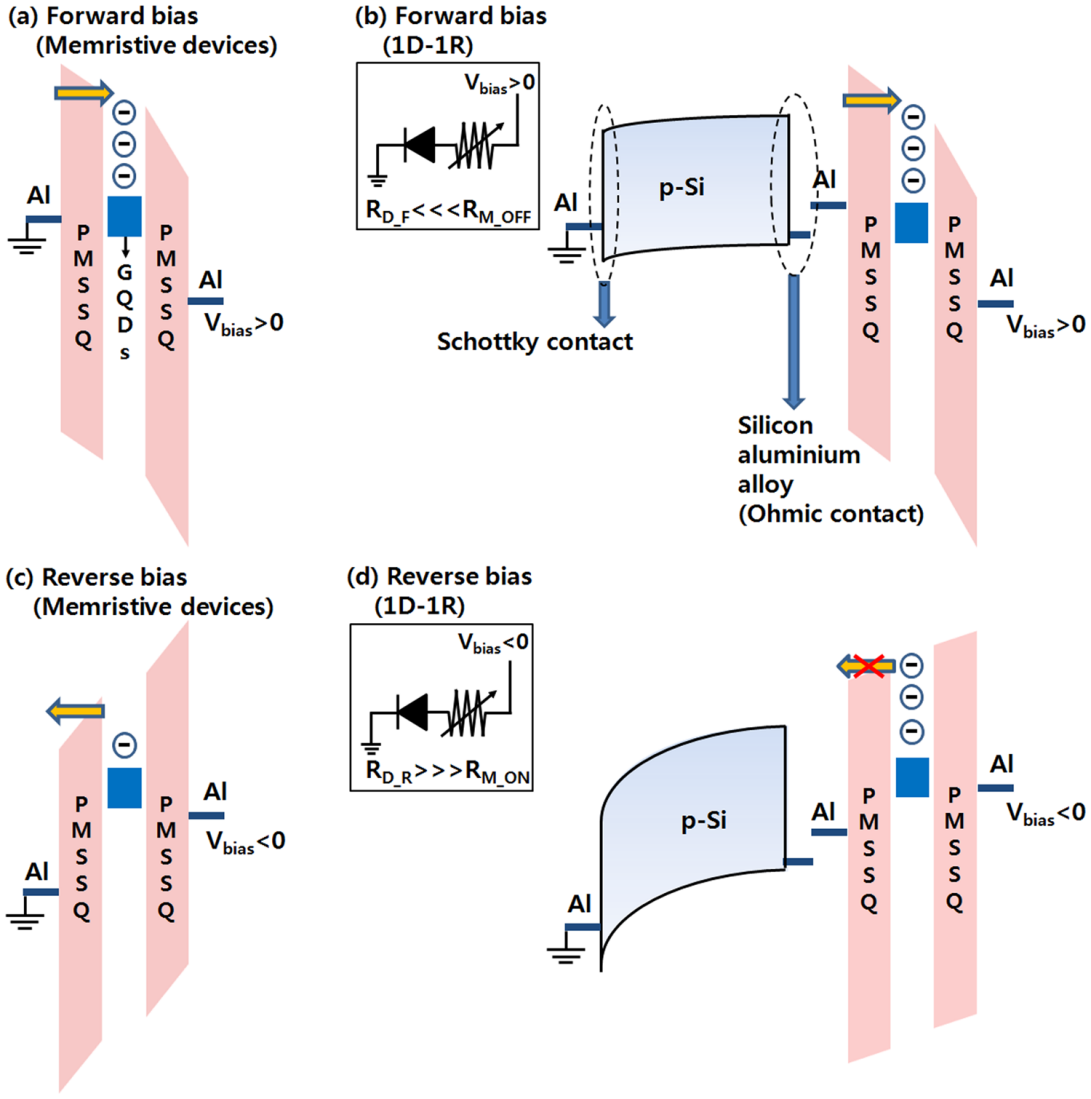
Schematic diagrams of the electronic structures corresponding to the operating mechanisms of the forward bias for the **(a)** Al/PMSSQ:GQDs/Al devices and the **(b)** Al/PMSSQ:GQDs/Al/p-Si/Al device and circuit diagrams (left insets) and reverse bias processes for the **(c)** Al/PMSSQ:GQDs/Al devices and the **(d)** Al/PMSSQ:GQDs/Al/p-Si/Al and circuit diagrams (left insets). The Ohmic contact is made with a silicon-aluminum alloy that forms in the middle of the Al/Si interface on the right in the figure, not with the p-Si; thus, the Fermi level will be as indicated in the figure. The Schottky contact is formed at the bottom of the Al/Si interface on the left in the figure.

Figure 6 shows schematic diagrams and circuit diagrams for the electronic structures of the Al/PMSSQ:GQDs/Al memristive device and the 1D–1R device corresponding to the operating mechanisms. The mechanism for the set/reset operation of the Al/PMSSQ:GQDs/Al memristive device is shown in Fig. 6(a,c). When a bias higher than the SET voltage is applied, in the OFF state, electrons are injected from the Al electrode into the lowest unoccupied molecular orbital (LUMO) of the PMSSQ via a Fowler-Nordheim (F-N) tunneling process^25^, and the GQDs act as electron trapping sites. As a result, space-charge formation due to electron trapping dominates the conduction process^26^. The curve fitting in Fig. 3(b) shows that the carrier transport in the ON state is due to Ohmic conduction, which is indicative of the formation of conducting paths in the active layer. That electrons captured in a polymer matrix can generate a high internal field, which can locally polarize the dielectric layer to form local conducting filaments in the insulating layer, is widely accepted; thus, such filaments are thought to be present in our active layer^27^. In our device, the GQDs act as traps in the PMSSQ matrix, and these traps can capture electrons and produce a high local internal field in the PMSSQ. Consequently, local conducting paths are formed in the PMSSQ layer under a high internal field. Thus, the current can flow more easily due to conductive filaments that are formed in the active layer. After the state transition from the OFF to the ON state, the current in the memristive device remains constant. When a high negative applied voltage (V > V_RESET_) is applied, the electrons trapped in the GQDs are de-trapped, which is known as an erasing process, as shown in Fig. 6(c). This causes a significant decrease in the current, and the device is switched to the OFF state. Even though the band diagram (Al/PMSSQ:GQDs/Al) shown in Fig. 6(a,c) is symmetric, the actual interface is more complex due to the different fabrication processes. For the middle Al/PMSSQ:QD interface, the PMSSQ:QD solution is spin-coated on the as-fabricated Al electrode. However, for the top Al/PMSSQ:QD interface, the Al electrode is deposited on the as-fabricated PMSSQ:QD layer by using thermal evaporation. As a result, the two interfaces may be different, leading to bipolar switching performances, which would be a good research topic for future work. Furthermore, the band diagrams shown in Fig. 6 are just schematics to help the reader to understand the proposed working mechanism, and the explanation of that mechanism in the ON state, we believe, is not affected by the number of electrons depicted in the schematic diagram.

The mechanism for the set operation of the 1D–1R device is shown in Fig. 6(b,d). When a positive voltage is applied to the device, the forward resistance of the diode (R_D_F_) is much smaller than that of the memory in the OFF state (R_M_OFF_), as shown in the inset of Fig. 6(b). Thus, the electrons emitted from the p-Si/Al Schottky diode are injected into the LUMO of the PMSSQ via the F-N tunneling process^25^, as shown in Fig. 6(b). Because the GQDs in the PMSSQ act as trapping sites, they capture the electrons emitted from the p-Si/Al. The electrons captured in the GQDs generate a local internal field in the PMSSQ layer, which results in a transition from an OFF to an ON state^27^. When a reverse voltage bias is applied, the reversed resistance of the diode (R_D_R_) is much larger than that of the memory in the ON state (R_M_ON_), as shown in the inset of Fig. 6(d). As a result, the actual potential applied to the memristive devices is much smaller than the resetting threshold voltage bias to the memory device. Thus, the device cannot be permanently switched to an OFF state, as shown in Fig. 6(d). As a result, the 1D–1R device is always in the ON state under the reading voltage.

## Conclusion

The I-V curves for the Al/PMSSO:GQDs/Al devices at room temperature showed that by adjusting the GQD concentration, we were able to maximize the memory margin of the devices at a GQD concentration of 10%, resulting in improved device performances. Using the measured electrical properties of the Al/PMSSQ:GQDs/Al devices, we fabricated WORM 1D–1R devices with an optimized structure of Al/PMSSQ:GQDs/Al/p-Si/Al, i.e., an optimal GQD concentration of 10% and an p-Si/Al Schottky diode, to prevent cross-talk in the Al/PMSSQ:GQDs/Al/p-Si/Al devices. The I-V curves for the WORM 1D–1R devices at room temperature demonstrated memory and rectification characteristics. The results of the retention measurements for the WORM 1D–1R devices demonstrated that they exhibited stable memory performance with retention times larger than 10^4^ s without any significant electrical degradation.
